# Task‐sharing in menstrual regulation services: Implementation efforts and lessons learned in Bangladesh

**DOI:** 10.1002/ijgo.13009

**Published:** 2020-07-31

**Authors:** Nadira Sultana

**Affiliations:** ^1^ Independent Consultant Dhaka Bangladesh

**Keywords:** Bangladesh, Family planning, Implementation, Menstrual regulation, Nonphysician providers, Task‐sharing

## Abstract

**Objective:**

To explore the strategies undertaken to decentralize menstrual regulation services and implement task‐sharing, including barriers and facilitators, with nonphysician providers in Bangladesh.

**Methods:**

We conducted a desk review of relevant policies and health service information from grey and published literature on task‐sharing in menstrual regulation services, plus stakeholder interviews with 19 representatives of relevant health organizations to investigate facilitators for and barriers to the implementation of task‐sharing of these services.

**Results:**

Task‐sharing in menstrual regulation began in 1979 as part of the national family planning program. The Ministry of Health and Family Welfare has guidelines for menstrual regulation services provided by a wide range of healthcare workers using manual vacuum aspiration and the medications misoprostol and mifepristone. Despite government approval, implementation of task‐sharing is challenging owing to lack of skilled providers, lack of facility readiness, and unmet need for family planning.

**Conclusion:**

The government needs to implement effective planning for skills building of nonphysician providers and ensuring facility readiness for provision of menstrual regulation services to reduce unsafe abortion in Bangladesh.

## Synopsis

Task‐sharing in menstrual regulation services is supported by the government in Bangladesh and associated policies and guidelines are available; however, shortage of skilled providers is a barrier.

## 1 Introduction

Bangladesh’s health indicators have improved since its independence in 1971 and the country has a proven track record of accomplishing the health‐related Millennium Development Goals (MDGs)^1^ However, a shortage of skilled staff has been identified as a key barrier to delivering quality care. Health regulations in Bangladesh permit “menstrual regulation” to “regulate the menstrual cycle when menstruation is absent for a short duration”.^2^ Any woman is eligible for this service if she misses her period. The government has included this service in the national family planning program as routine care,^2^, ^3^ whereas abortion is only permitted—at any gestational age—to save the life of the woman according to the Bangladesh Penal Code (Act XLV, October 6, 1860).^4^ Menstrual regulation can be performed up to 12 weeks without legal restriction.^3^, ^5^

Nonphysician providers have been involved in providing menstrual regulation services since the program began in 1979. Nurses, paramedics, and family welfare visitors (FWVs) trained in these services are permitted to perform menstrual regulation up to 10 weeks of gestation by manual vacuum aspiration (MVA) both in public and private facilities. In addition, medical doctors can perform menstrual regulation up to 12 weeks of gestation within the legal framework and guidelines from the Ministry of Health and Family (MOHFW).^3^ Date of last menstrual period is used to determine gestational age.

To provide context, FWVs are frontline providers responsible for a range of health and family planning services. The minimum educational requirement for an FWV is secondary school certification passed in the country’s education system and, after enrollment, 18 months of residential training from specific FWV training institutes. FWVs are stationed primarily in remote areas at union health and family welfare centers (UHFWCs) throughout Bangladesh, under the Directorate General of Family Planning (DGFP) after completion of training.^6^ Training in menstrual regulation with MVA is provided separately and lasts 3 weeks.^3^

The situation in Bangladesh is an example of where task‐sharing has been accepted for menstrual regulation services without dissent from stakeholders. Regrettably, several barriers continue to hamper implementation of services even though favorable policy and national guidelines exist. The aim of the present study was to explore the strategies undertaken to decentralize menstrual regulation service delivery and implement task‐sharing, including the barriers and facilitators, with nonphysician providers in Bangladesh.

## 2 Materials and Methods

We conducted a desk review, from May to November 2018, of relevant policies and health service information from the grey and published literature on task‐sharing for menstrual regulation services in Bangladesh, since its initiation in 1979. Literature on task‐sharing in overall health care and information on policies and practices applicable to nonphysician providers in maternal care and their implications were also reviewed. Finally, we sought information on the prevalence of unsafe abortion and its effect on morbidity and mortality.

The most recent Bangladesh Maternal Mortality Survey (BMMS) 2016 was taken into consideration. This report showed that the maternal mortality ratio (MMR) stalled between 2010 and 2016 at 196 maternal deaths per 100 000 live births. The report also revealed that the MMR increased between 2010 and 2016 as a result of unsafe abortion, from two to 15 maternal deaths attributed to this cause per 100 000 live births; and that abortion‐related maternal deaths peaked among women aged 30–34 years.^7^ The Guttmacher Institute reported that inadequately skilled providers and a lack of facility readiness across the country were contributing factors.^8^

Guidelines from the MOHFW on the national menstrual regulation program approve task‐sharing to midlevel healthcare providers but only up to a specific time limit of gestational age.^3^ The World Health Organization (WHO) has technical guidelines on health worker roles for providing safe abortion and postabortion care.^9^ WHO has promoted task‐sharing by providing different technical directions and recommendations for global implementation.^10^

We conducted in‐person structured key informant interviews with 19 senior‐level representatives from governmental and nongovernmental organizations (NGOs) providing menstrual regulation services to understand their perspectives on the facilitators for and barriers to the implementation of task‐sharing for menstrual regulation services in Bangladesh. Interviews were conducted in June and July 2018. Procedures for scheduling in‐person meetings and publishing the information provided were agreed. Participants signed informed consent forms. From MOHFW, both the program manager of the Directorate General of Health Services (DGHS) and the DGFP were interviewed to obtain the government’s view on the current situation regarding menstrual regulation services. Interviews were held with two top‐level managers from one public facility and training center: the Mohammadpur Fertility Services and Training Centre under the DGFP. Three program managers from three respective NGOs took part in interview sessions: Marie Stopes Bangladesh (MSB), Reproductive Health Services Training and Education Programme (RHSTEP), and the Association for Prevention of Septic Abortion, Bangladesh (BAPSA). One representative from the midwifery program and one representative from the Obstetrical and Gynecological Society of Bangladesh (OGSB) were also interviewed. Hence, consultations with the 19 key informants provided information on the country’s health system in relation to menstrual regulation services and its policy context.

## 3 Results

### 3.1 Availability of menstrual regulation services

In Bangladesh, menstrual regulation services are supposed to be available at all health facility levels, including medical institutions; medical college hospitals; district hospitals; Maternal and Child Health and Family Planning Centers; upazila health complex clinics (a public health facility at subdistrict level; 492 in the country at December 2017); Maternal and Child Welfare Centers; Union Health and Family Welfare Centers; and government‐approved private and NGO health facilities.^3^

Specific training is mandatory for those providing menstrual regulation services using MVA, including medical doctors. The government allows trained midlevel health workers, such as FWVs, female paramedics, female subassistant community medical officers (SACMOs), NGO paramedics, and nurses to perform menstrual regulation using MVA up to a maximum of 10 weeks of gestation. The same procedure is extended to a maximum of 12 weeks of gestation when performed by medical doctors. Menstrual regulation with medication (misoprostol and mifepristone) is allowed up to 9 weeks from the last menstrual period by nonphysician providers who have received appropriate training. A number of government and NGO training institutes provide 3 weeks of intense training on menstrual regulation with MVA for nonphysician service providers and orientation on using misoprostol and mifepristone in addition to the training offered by medical college hospitals.^3^, ^5^

Menstrual regulation can be provided as an outpatient procedure at government service centers, government‐approved NGOs, and private institutes/hospitals/clinics that have trained and skilled menstrual regulation service providers, and available materials and equipment. Menstrual regulation services are free of charge in all government facilities.

Guiding principles exist for service providers to respect patients’ right to confidentiality. Providers must promote women’s rights to dignity and autonomy and must uphold conscientious objection and refer patients, if necessary. Furthermore, counseling must be offered before, during, and after the procedure. Consent must be obtained from a woman before she can undergo MVA. In special cases, such as mentally handicapped or younger women, the consent of the guardian/relative/husband must also be obtained.^3^ Use of medication for menstrual regulation in the national family planning program has been approved by the National Technical Committee of the DGFP. The combination of mifepristone and misoprostol is available as a kit and several pharmaceutical companies produce the drugs. DGFP wants to ensure that this service is available at all approved health facilities where MVA‐trained providers and materials are available.^5^

Bangladesh’s cognizance of task‐sharing for safe abortion has been discussed in many national and international forums. Under MOHFW, the DGFP is the prime organization supporting menstrual regulation services as part of the national program in Bangladesh. The majority of healthcare workers designated to provide menstrual regulation services are FWVs—the major service provider for the DGFP, even in remote rural areas. Female SACMOs under the DGHS who have received training can provide menstrual regulation services at the community level. The Mohammadpur Fertility Services and Training Centre is one of the largest facilities under the DGFP, providing services and training providers. UNFPA, Ipas, and other partners also support training of field‐level staff in both public and private sectors.

### 3.2 Implementation of task‐sharing and barriers to menstrual regulation services

Despite existing favorable policy, a recent survey suggested that 7% of maternal deaths are caused by unsafe abortion. Several issues are associated with unsafe abortion, which increased between 2010 and 2016.^7^ The Bangladesh Population Policy 2012 prioritizes reducing the total fertility rate, increasing availability of family planning methods, promoting safe motherhood, achieving gender equity, harnessing the population’s human resource capacity, and ensuring easy access to reproductive health information and services, which includes menstrual regulation services.^11^ Implementing task‐sharing for nonphysician health workers is crucial for successful implementation of the national population policy, particularly in rural areas where FWVs are the first contact for family planning and reproductive health services. In reality, doctors are rarely available for family planning and reproductive health services in rural areas.

The Bangladesh Demographic and Health Survey (BDHS) 2014 reported a contraceptive prevalence rate of 62% and unmet need for family planning of 12%, which has been unchanged from 2011.^12^ Challenges exist in terms of family planning discontinuation rate, suboptimal use of methods, lack of availability of all methods, lack of trained staff, and unmet need. These limitations are reflected in the statistics that an estimated 1.3 million menstrual regulation services and illegal abortion procedures are conducted annually.^8^

The Guttmacher Institute reported that the number of facilities providing menstrual regulation services reduced between 2010 and 2014.^8^ Nationally, only 53% of public sector facilities permitted to provide menstrual regulation services actually did so in 2014 (down from 66% in 2010); in the same period this proportion was only 20% among private sector facilities (down from 36% in 2010).^8^ Concurrently, the BDHS 2014 reported that public sector facilities were the major provider (48%) of menstrual regulation services among ever‐married women who had used the service in the last 3 years, followed by private medical facilities (33%), and NGO facilities (6%).^12^

BDHS 2014 further reported that there are gaps in services and information on the availability of menstrual regulation at the community level.^12^ Many women are unaware of the availability of safe and legal menstrual regulation services.^8^ The need is greatest among marginalized populations: women residing in urban slums, the rural poor, and young girls under 18 years who are married at an early age. Notably, more than half of all girls are married before the legal age of 18 years in Bangladesh, which has some of the highest rates of child marriage worldwide.^13^ When access to formally established menstrual regulation services is limited, women tend to seek aid from other sources such as untrained and illegal providers, often under unsafe conditions.^8^

The literature review revealed that total demand for family planning (the sum of total unmet need and total contraceptive use) in Bangladesh is 74%.^12^ The concern is that women who have an unmet need for family planning might resort to unsafe abortion. Another study reported that, in 2014, some 2.8 million pregnancies—48% of all pregnancies—were unintended. Unsafe abortion and menstrual regulation procedures were associated with three‐fifths of unintended pregnancies.^8^ Therefore, the government and NGOs are working together to add additional cadres of nonphysician health workers able to provide menstrual regulation services; for example, recently recruited midwives at upazila health complexes.

A recent study found that one‐third of facilities that could provide menstrual regulation services lacked either trained staff or equipment, or both.^14^ At subdistrict (upazila) level and below, 92% of providers do not provide menstrual regulation services because of lack of training.^10^ According to WHO criteria, only 22% of all facilities are ready to provide quality family planning services.^15^ Half of NGO facilities and 5% of private sector facilities are prepared and equipped to offer quality family planning services.^15^

Lack of available services and lack of awareness of legal services have resulted in the clandestine practice of unsafe abortion, which remains a threat to maternal health. The lack of a sufficient health workforce is a critical issue in terms of the imbalance in mix of skills, maldistribution of skilled staff, work environments without basic facilities, and weak knowledge base due to untrained staff.^15^ Considering the total population of Bangladesh (more than 160 million people), the lack of available human health resources underscores the inadequacy of the health system at every level. There are only 0.58 health workers in Bangladesh for every 1000 population, including FWVs and SACMOs, and only 0.3 nurses and midwives for every 1000 population. The doctor to population ratio is 1:1500 in urban areas, whereas it is 1:15 000 in rural areas. A total of 4898 FWVs were in service according to human resource data available from MOHFW in 2014.^15^ These figures led to the inclusion of Bangladesh in the WHO list of 57 countries facing an acute crisis in human health resources.^16^ Qualified midwives have recently begun working in some health centers, which was absent before 2010.^18^

Training in MVA procedures to ensure quality of menstrual regulation care is important for all nonphysician health workers. However, the government does not have adequate budget allocation for training staff in this procedure and there is limited funding support from donors. Additionally, the training program has yet to be institutionalized in the course curriculum of pre‐service and in‐service, or continuing education. Although menstrual regulation is an approved service, the capacity building initiative has been absent in recent years. Again, lack of equipment is a problem. Single‐valve Karman syringes and Ipas MVA Plus aspirators are procured by the DGFP but are not available in the facilities under the DGHS, such as university teaching hospitals, medical college hospitals, district hospitals, or medical institutes.^18^

Low level of education and lack of awareness about family planning among women of reproductive age and their partners may result in unintended pregnancy and clandestine abortion. Only 9% of women who used menstrual regulation services reported getting the information from trained family planning providers. In some cases, service providers have turned away women seeking menstrual regulation services.^8^ An estimated 27% of women seeking such services at public and private facilities were rejected by providers as they exceeded the approved limit of 10–12 gestational weeks^8^; this was the most common reason given by providers for rejecting women seeking menstrual regulation services.^8^ The community is largely unaware of the availability of menstrual regulation services at nearby facilities.

In 1987, two committees were established for the management of menstrual regulation training and services: The National Technical Committee (NTC) and the Coordination Committee of Menstrual Regulation Agencies in Bangladesh (CCMRB).^3^ The CCMRB is no longer active and its functions have been stopped. In 1990, the Technical Advisory Committee was formed headed by the DGFP. Neither of the current committees is functioning optimally; if they were it would enable improvements in access to and availability of menstrual regulation services.

The Fourth Health Population Nutrition Sector Program (HPNSP) 2017–2022 was designed to incorporate strategies and activities to improve access to quality health care, equity, and universal health coverage.^19^ Unfortunately, menstrual regulation care is not specifically mentioned either in the DGHS or in the DGFP operation plans in the Fourth HPNSP, although it is incorporated into family planning and maternal healthcare programs. Furthermore, capacity building for staff delivering menstrual regulation services has not been captured in the operation plans.

## 4 Discussion

Bangladesh is an example of a country in which task‐sharing for provision of menstrual regulation services has government support. Nonphysician providers are permitted to provide menstrual regulation services at different health facility levels both in urban and rural areas. However, constraining and weakening factors limiting service provision include human resource management, quality of care, and logistic support. In many facilities providing menstrual regulation services, infection prevention protocols and standard service delivery practice were inadequate.^8^ The disparities between government policies and implementation of task‐sharing for provision of menstrual regulation services is evident. The lack of skilled service providers must be resolved, and other barriers need to be addressed.

DGHS is the largest network of providers and facilities. It has the scope to expand task‐sharing for menstrual regulation services across the facilities among nurses, midwives, female SACMOs, qualified community health clinic providers, and community skilled birth attendants after providing required training and skills building. It should be encouraged to do this. Pre‐service education and training are also important. Bangladesh must design and implement various approaches for reducing unwanted fertility to address the needs of specific population groups. For example, regional service packages can be developed to strengthen quality family planning and menstrual regulation service delivery in low‐performing and hard‐to‐reach areas, including urban slums. To optimize task‐sharing, staff capacity and readiness of the facilities need to be ensured, along with supervision and monitoring. Both the DGHS and DGFP need to allocate funds for staff capacity building for successful task‐sharing among nonphysician health workers.

In conclusion, a wide range of nonphysician service providers are permitted to provide menstrual regulation services in Bangladesh and the policies and guidelines are favorable. The barriers identified are primarily at implementation level due to lack of skilled providers, materials, or logistics. It is therefore important to make a comprehensive operational plan including financial allocation, training, monitoring, and supervision. Capacity building of providers is fundamental for successful implementation of task‐sharing along with consistent supplies of required resources. Monitoring and supervision should be integrated with operational plans. If the government makes solid efforts to implement its menstrual regulation program by nonphysician service providers, per the guidelines, Bangladesh would be an ideal model for task‐sharing in abortion care.

## Conflicts of Interest

The author has no conflicts of interest.

## References

[1] General Economics Division (GED), Bangladesh Planning Commission, Ministry of Planning, Government of the People’s Republic of Bangladesh . Sustainable Development Goals: Bangladesh Progress Report 2018 https://www.undp.org/content/dam/bangladesh/docs/Publications/Pub-2019/SDGs-Bangladesh_Progress_Report%202018%20(1).pdf. Accessed August 20, 2019.

[2] Women on Waves . Bangladesh Abortion Law [website]. https://www.womenonwaves.org/en/page/4866/bangladesh–abortion-law. Accessed November 2, 2019.

[3] Directorate General of Family Planning, World Health Organization Country Office for Bangladesh, Kingdom of the Netherlands . Bangladesh National Menstrual Regulation Services Guidelines. https://abortion-policies.srhr.org/documents/countries/05-BANGLADESH-NATIONAL-MENSTRUAL-REGULATION-SERVICES-GUIDELINES.pdf. Accessed August 30, 2019.

[4] Rahman A , Katzive L , Henshaw SK . A Global Review of Laws on Induced Abortion, 1985–1997. Int Fam Plann Persp. 1998;24:56–64. 14627052

[5] MCH‐Services Unit, Directorate General of Family Planning, Ministry of Health and Family Welfare . Bangladesh National Service Delivery Guideline on Menstrual Regulation with Medication (MRM). Dhaka: MCH‐Services Unit, Directorate General of Family Planning; 2015 https://abortion-policies.srhr.org/documents/countries/06-BANGLADESH-NATIONAL-SERVICE-DELIVERY-GUIDELINE-ON-MENSTRUAL-REGULATION-WITH-MEDICATION.pdf. Accessed July, 2019.

[6] Mridha MA , Khan NA . An Appraisal of the Institutional Training Arrangement for Community Health Workers in Bangladesh. https://www.who.int/hrh/en/HRDJ_4_2_04.pdf. Accessed October 20, 2019.

[7] National Institute of Population Research and Training, International Centre for Diarrhoeal Disease Research, Bangladesh, and MEASURE Evaluation . Bangladesh Maternal Mortality and Health Care Survey 2016: Preliminary Report. Dhaka, Bangladesh, and Chapel Hill, NC, USA: NIPORT, icddr,b, and MEASURE Evaluation; 2017.

[8] Hossain A , Maddow‐Zimet I , Ingerick M , Bhuiyan HU , Vlassoff M , Singh S . Access to and Quality of Menstrual Regulation and Postabortion Care in Bangladesh: Evidence from a Survey of Health Facilities, 2014. 2017 https://www.guttmacher.org/report/menstrual-regulation-postabortion-care-bangladesh. Accessed July 27, 2019.

[9] World Health Organization . Health Worker Roles in Providing Safe Abortion Care and Post‐abortion Contraception. Geneva: WHO; 2015. 26401543

[10] Ganatra B . Health worker roles in safe abortion care and post‐abortion. Lancet Glob Health. 2015;3:e512–e513. 26231424 10.1016/S2214-109X(15)00145-X

[11] Ministry of Health and Family Welfare, Peoples Republic of Bangladesh . Bangladesh Population Policy 2012. Dhaka: Ministry of Health and Family Welfare; 2012.

[12] National Institute of Population Research and Training (NIPORT), Mitra and Associates, and ICF International . Bangladesh Demographic and Health Survey 2014. Dhaka, Bangladesh, and Rockville, Maryland: NIPORT, Mitra and Associates, and ICF International; 2016.

[13] UNFPA, Bangladesh . Child marriage [Website]. https://bangladesh.unfpa.org/en/topics/child-marriage-2. Accessed October 20, 2019.

[14] National Institute of Population Research and Training (NIPORT), Associates for Community and Population Research, ICF . Bangladesh Health Facility Survey 2017. Dhaka, Bangladesh: NIPORT, ACPR, and ICF; 2018.

[15] Ministry of Health and Family Welfare, Government of Peoples Republic of Bangladesh . HRH Data Sheet. Dhaka, Bangladesh: Human Resource Management Unit, Ministry of Health and Family Planning; 2014.

[16] World Health Organization, Bangladesh . Bangladesh Health Workforce Strategy 2015: On the Move [Website]. http://www.searo.who.int/bangladesh/news/BAN_HTS/en/. Accessed July 30, 2019.

[17] UNFPA, Bangladesh . Midwifery [Website]. https://bangladesh.unfpa.org/en/topics/midwifery-0. Accessed October 20, 2019.

[18] Begum F , Zaidi S , Fatima P , Shamsuddin L , Anowar‐ul‐Azim AK , Begum RA . Improving manual vacuum aspiration service delivery, introducing misoprostol for cases of incomplete abortion, and strengthening postabortion contraception in Bangladesh. Int J Gynecol Obstet. 2014;126(Suppl):S31–S35. 10.1016/j.ijgo.2014.03.004 24792403

[19] Ministry of Health and Family Welfare, Peoples Republic of Bangladesh . Health, Nutrition and Population Strategic Investment Plan 2016‐2021. Dhaka: Ministry of Health and Family Welfare; 2016.

